# Adsorption of Forever Chemical Pollutants: The Physical Chemistry of PFAS Near Surfaces

**DOI:** 10.1002/advs.75893

**Published:** 2026-06-17

**Authors:** Nada Ben Amor, Daniela Bauer, Benjamin Braconnier, Benoit Coasne

**Affiliations:** ^1^ University Grenoble Alpes, LIPhy Grenoble France; ^2^ IFP Energies nouvelles Rueil‐Malmaison France; ^3^ University Grenoble Alpes, CNRS, LIPhy Grenoble France; ^4^ Institut Laue Langevin Grenoble France

**Keywords:** adsorption thermodynamics, mixed‐order kinetics, PFAS

## Abstract

Due to their persistence, per‐ and polyfluoroalkyl substances (PFAS) raise concerns that challenge current water remediation strategies. While adsorption‐based solutions appear promising, their development is limited by knowledge gaps on PFAS behavior near solid surfaces. This review provides a state of the art on the theoretical and experimental aspects of PFAS adsorption. By adopting a fundamental physical chemistry standpoint, we report recent advances in understanding PFAS adsorption under relevant thermodynamic and chemical conditions. First, we introduce the fundamental interactions involved in the adsorption of individual molecules on surfaces, before addressing collective behaviors such as self‐aggregation, ionic bridging, and competition with organic matter. We also present the thermodynamics and kinetics of PFAS adsorption using classical models. In particular, an accurate definition of the adsorption and desorption rates is given along with the key factors determining the kinetic order (i.e., first‐, second‐ or mixed‐order). Both batch and kinetic adsorption experiments are analyzed to identify the role of surface and PFAS structure and chemistry. Then, we evaluate how environmental factors (pH, salinity, copollutants, organic matter) impact adsorption. We conclude this review by identifying the perspectives in this field.

## Graphical abstract

Current adsorption‐based remediation techniques for removing per‐ and polyfluoroalkyl substances (PFAS) from water are limited by knowledge gaps on PFAS behavior near solid surfaces. This review provides a state of the art on theoretical and experimental aspects of PFAS adsorption. By adopting a fundamental physical‐chemistry standpoint, we report recent advances in PFAS adsorption under relevant thermodynamic and chemical conditions.

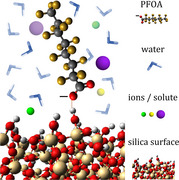

## 1 Introduction

**FIGURE 1:**
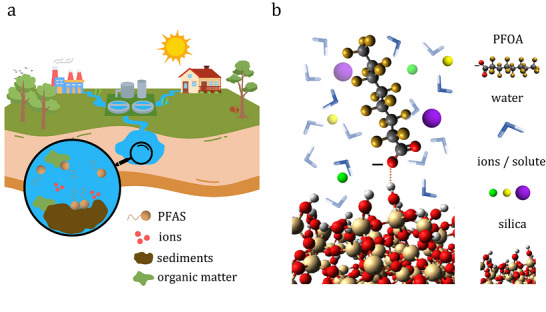
(a) Cartoon illustration of the path followed by PFAS in the environment. As shown in the zoom, PFAS can interact with coexisting electrolytes and organic matter and adsorb on solid surfaces such as natural clays and sedimentary rocks. (b) Microscopic illustration of PFAS molecules adsorbed on a hydroxylated silica surface. Here, a typical adsorption scenario is represented with a dissociated perfluorooctanoic acid (PFOA) being adsorbed on a protonated silica surface.

Perfluoroalkyl and polyfluoroalkyl substances (PFAS) constitute a family of synthesized compounds receiving tremendous attention [1, 2], as they are considered a major threat to the environment and human beings [3, 4, 5]. In particular, recent publications have raised concerns about the impact of PFAS on human health [3, 4, 5, 6, 7, 8, 9, 10]. Further reading on these aspects includes a review published in 2021 on assessing the ecological risks of PFAS [11]. To be considered as PFAS, a molecule should possess at least one perfluoroalkyl moiety – $C_{n}F_{2n+1}$ – (where n typically ranges from 4 to 14) [12]. Within this class, an important subgroup consists of fluorinated surfactants composed of a hydrophilic head and a hydrophobic tail made of fluorine atoms [13, 14]. Owing to their unique properties, PFAS are used in industry for anti‐adhesive applications, heat‐resistant materials, waterproofing, and in various consumer products (e.g. cosmetics, kitchenware, furniture, food containers, etc.) [2, 15]. They are also of practical importance as materials for outdoor activities (e.g.skiing, fishing) [16, 17, 18]. With their broad usage, large amounts of PFAS emitted from industrial plants or domestic activities are found in wastewater plants [19] and landfill leachates [20, 21]. Eventually, PFAS get dispersed across numerous environmental media, including groundwater (Figure 1a) [22], river water [23, 24, 25], marine water [26], microestuaries (i.e., small water bodies of less than 0.5${\mathrm{km}}^{2}$) [27], soils [16, 28], and leachates [20, 29]. The occurrence of PFAS molecules in natural media has been monitored by several research instances or groups (e.g. [30, 31, 32]) and multiple sampling campaigns have reported the presence of PFAS in treated drinking water [33], and in food products [34]. In addition, some PFAS were detected in 100% of tested children and adults during a French biomonitoring program (Esteban) [35]. Studies on fetal exposure have further confirmed the presence of both legacy and emerging PFAS compounds in serum, placenta, and meconium samples [31].

A specific threat that represents the contamination of water and natural environments by PFAS is their high stability [36]. This feature renders conventional degradation processes inefficient and, hence, points to the need to develop alternative removal strategies [37, 38, 39, 40]. The stability of these compounds arises from the intimate nature of the C‐F bonds, which render fluorinated chains highly stable (especially for long‐chain PFAS) [36]. In particular, the chemical stability of fluoroalkyl chains makes PFAS more resistant to chemical decomposition [41] (e.g. hydrolysis, photolysis, and oxidation) and to biological degradation (e.g. microbial [42, 43] and enzymatic degradation [44]). In contrast, adsorption‐based remediation treatments appear to be a viable solution for PFAS removal [45]. Indeed, considering the richness of molecular interactions involved at PFAS/solid interfaces, adsorption can be tuned to achieve efficient remediation in various thermodynamic and physicochemical conditions. Nevertheless, in light of the current state‐of‐the‐art, designing cost‐effective adsorption materials and processes with high selectivity for PFAS molecules is challenging. In particular, a better understanding of the fundamental mechanisms underlying adsorption is needed to open novel perspectives for effective strategies. The development of potential water treatment protocols also requires acquiring a deeper understanding of the behavior of PFAS under realistic conditions. Furthermore, closing knowledge gaps in this field would allow the development of tools to quantify the environmental impact of PFAS via adsorption mechanisms.

From a fundamental perspective, the main physicochemical features of PFAS adsorption can be described as follows. We consider the situation schematically depicted in Figure 1b, which provides a molecular view of the problem of PFAS adsorption at a mineral surface (here, hydroxylated silica is used as a prototypical example). First, in conventional aqueous environments, most commonly encountered PFAS (e.g. perfluoroalkyl acids) exist in a dissociated form: the head is negatively charged (therefore hydrophilic) while the tail is apolar (therefore hydrophobic) [46]. Consequently, owing to their amphiphilic nature, PFAS experience both ionic and non‐ionic interactions whose competition can lead to a wide range of adsorption behaviors. In particular, depending on the experimental conditions, those compounds can bind to positively charged surfaces and/or to hydrophobic surfaces through dispersive interactions. As will be discussed at length in this review, in most practical situations, both ionic and non‐ionic interactions drive PFAS adsorption on a given surface. Moreover, considering their tendency to aggregate in aqueous solutions [47], understanding the individual molecular interactions at play upon PFAS adsorption is insufficient as their collective behavior becomes important near surfaces (i.e., when PFAS concentration is above a certain threshold). The role of the water medium and its physicochemical features (pH, temperature, salinity, presence of other contaminants and/or organic matter) is also an essential parameter to consider. In particular, depending on the origin of the water medium (e.g. river, marine or groundwater), PFAS molecules coexist and interact with many other chemical substances such as electrolytes, natural and/or effluent organic matter, heavy metals, etc. As a result, besides the intrinsic nature of the PFAS molecules and the adsorbing surface, the presence of other chemical species as well as the salinity and the pH levels of the environment determine to a large extent the observed specific PFAS adsorption behavior. Finally, given their surfactant‐like nature, the temperature of the medium influences the state in which PFAS are found in solution (monomers, liquid crystals, or micelles). Therefore, better characterizing their solubility line and its temperature dependence near surfaces is essential – especially for compounds exposed to a wide range of environmental temperatures.

This review aims to provide a comprehensive description of the fundamentals of PFAS adsorption thermodynamics and kinetics – including the complex phenomena that may arise from the intrinsic and rich nature of PFAS in combination with the wide variety of realistic environmental factors mentioned above. While many reviews are already available on the presence and fate of these “forever chemicals” [41, 48, 49], they mostly adopt an environmental engineering or sanitary/health point of view by focusing on the threats and growing concerns that cause PFAS. While the scope of this review is limited to PFAS molecules, which typically possess a molecular length of a few nanometers, the richness and the heterogeneity of PFAS compounds suggest the possibility to generalize and extend the picture detailed in this review to other chemicals – especially, surfactants as they share similarities such as amphiphilicity, aggregation with collective effects, variable hydrophilicity/hydrophobicity, etc.

## 2 Fundamentals of PFAS Adsorption Thermodynamics and Kinetics

### 2.1 Adsorption Mechanisms and Physical Interactions

#### 2.1.1 Molecular Interactions

Molecular interactions involve a broad range of physical interaction potentials corresponding to both Coulomb and dispersive/repulsive interactions. These contributions can be described as pair interactions between atoms in PFAS, an atom in a PFAS and a surface atom, and an atom in a PFAS with a coexisting compound in the solution (e.g. ionic species, non‐ionic organic matter, heavy metal ions).

From a fundamental viewpoint, the physical interactions involved in molecular processes lead to a potential energy U which contains all pairwise molecular interactions. Let us consider two atomic sites i and j belonging to two different molecules and separated by a distance r. The pairwise potential energy $U_{ij}\left(r\right)$ arising from the electrostatic (electron level) interactions between these two atoms writes [50, 51]:

(1) $$\begin{array}{c} \begin{array}{cc} U_{ij}\left(r\right) & =A^{ij}\exp\left(-b^{ij}r\right)-\frac{C_{6}^{ij}}{r^{6}}-\frac{C_{8}^{ij}}{r^{8}}-\frac{C_{10}^{ij}}{r^{10}}-\cdots +\frac{q_{i}q_{j}}{4\pi \varepsilon_{0}r}\\ & ∼4\varepsilon_{ij}\left[{\left(\frac{\sigma_{ij}}{r}\right)}^{12}-{\left(\frac{\sigma_{ij}}{r}\right)}^{6}\right]+\frac{q_{i}q_{j}}{4\pi \varepsilon_{0}r} \end{array} \end{array}$$

where the parameters $A^{ij}$, $b^{ij}$, $C_{6}^{ij}$, $C_{8}^{ij}$, $C_{10}^{ij}$, and $\sigma_{ij}$ depend on the nature of the atomic sites i and j, while $\varepsilon_{0}$ is the vacuum permittivity. The first term corresponds to Pauli principle as it describes – in a classical fashion – the repulsive interaction between electrons belonging to different atoms. The series $C_{2n}/r^{2n}$ with n=3,4,… account for dispersive interactions between fluctuating (i.e. non‐permanent) electrostatic dipoles, quadrupoles, etc. formed in the electron clouds of the two atoms. The third term corresponds to the Coulomb interaction between the partial charges $q_{i}$ and $q_{j}$ (if any) carried by the atoms.

**FIGURE 2:**
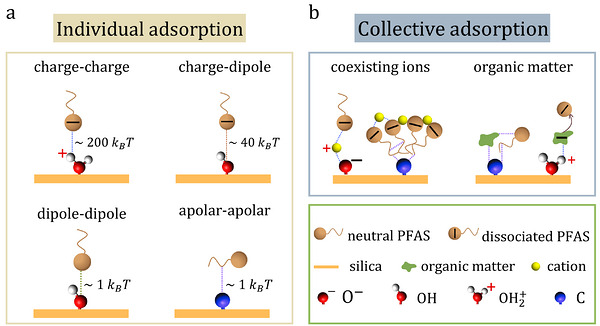
(a) Molecular interactions involved in PFAS adsorption reduce to four fundamental interactions: charge–charge, charge–dipole, dipole–dipole, and apolar–apolar interactions, with binding energies (in $k_{B}T$) that are calculated for molecules interacting in vacuum – discarding any solvent effect that would screen the molecular interactions through its dielectric constant. In aqueous solutions, those values should be rescaled by taking into account the dielectric constant of water (ε ∼ 78). (b) In realistic aqueous environments, PFAS molecules coexist with other species such as ions and organic matter, and collective adsorption processes also occur. The latter involve the same interactions as those presented in (a) but the resulting mechanisms are more complex.

The sum of the repulsive and dispersive interactions are known as the Buckingham potential (in practice, the series $C_{2n}/r^{2n}$ in the dispersion contributions is usually truncated after the term in $r^{-6}$ as the other terms decay extremely fast with distance r). As shown in the second equality of the equation above, the Buckingham potential is usually replaced by the Lennard–Jones potential which assumes that the exponentially decaying repulsive contribution Aexp(−br) can be replaced by $U∼r^{-12}$, while the dispersion term scales as $r^{-6}$. Equation (1) provides a cornerstone to describe all molecular interactions in PFAS adsorption and beyond. While repulsion does not contribute to adsorption (it simply describes that the adsorbed molecule does not penetrate into the solid), the dispersive and Coulomb interactions are responsible for adsorption. Dispersive interactions lead to apolar–apolar interactions – often referred to as hydrophobic interactions in the context of PFAS adsorption. Instead, depending on the nature of the interacting species, the electrostatic interactions can be recast in terms of charge–charge, charge–dipole or dipole–dipole interactions. Figure 2a illustrates the different molecular interactions involved in PFAS adsorption: (1) charge–charge, (2) charge–dipole, (3) dipole–dipole, and (4) apolar–apolar. In what follows, we detail these molecular interactions and provide their characteristic energy E and range $E∼r^{n}$ (expressed as the typical scaling involving the distance r and an exponent n).

##### 2.1.1.1 Charge – charge interactions

One of the main molecular interactions ruling the adsorption of charged PFAS is the attractive electrostatic interaction between their charged headgroup and the surface partial charge. Repulsive electrostatic interactions between charged headgroups also play a role as they impact the orientation and distance between PFAS molecules adsorbed on the surface. This interaction scales as $E∼r^{-1}$ with a typical energy E∼100−300 $k_{B}T$ [52]. While the charge‐charge interaction is strong and long‐ranged, it depends on external factors such as pH, presence of coexisting ionic species, etc.

##### 2.1.1.2 Charge – dipole interactions

PFAS adsorption also involves electrostatic interactions between a charged molecule and a neutral but polar molecule. For instance, hydrogen bonding may arise between a neutral polar adsorption site (e.g. OH group on a silicate surface) and a charged PFAS. The typical energy associated with this physical binding is $E∼40k_{B}T$ and scales as $E∼r^{-2}$ [52].

##### 2.1.1.3 Dipole – dipole interactions

Another electrostatic interaction involved in PFAS adsorption is the dipole–dipole interaction between a PFAS and the surface. This molecular interaction has a magnitude $E∼1k_{B}T$ and scales as $E∼r^{-3}$. This specific interaction is involved in the hydrogen bond formed between a polar surface and a non‐dissociated (i.e. neutral) PFAS, but it can often be neglected because PFAS adsorption competes with that of water [53].

##### 2.1.1.4 Apolar – apolar interactions

The dispersive interactions between apolar molecules are of order $E∼1k_{B}T$ and scale as $E∼r^{-6}$. Their contribution to adsorption increases with the chain length l of the PFAS molecule and it is particularly relevant as it can counterbalance repulsive Coulomb interactions, therefore allowing adsorption to occur even when unfavorable conditions apply (e.g. at very low pH when both the adsorbing PFAS and the surface can be negatively charged).

#### 2.1.2 Underlying Mechanisms

When combined together in a complex many‐body system involving a solvent, PFAS molecules and other chemical compounds, the fundamental repulsive, dispersive and electrostatic interactions lead to effective interactions. The latter are coined as “*hydrophobic interactions*” or “*electrostatic interactions*” depending on the dominant adsorption molecular forces. While hydrophobic interactions arise from the dispersive interactions, the electrostatic interactions arise from the Coulomb interaction potential. Electrostatic interactions are generally invoked to describe the direct interaction between PFAS charged headgroup and the surface charged sites. Such Coulomb interactions are also involved in the repulsive interaction between charged headgroups. As for the hydrophobic interactions, they arise between the PFAS hydrophobic tail and the solid hydrophobic regions as well as between two hydrophobic PFAS chains. PFAS hydrophobicity is also giving rise to a repulsive force between water molecules and PFAS tails, which favors PFAS adsorption at the solid surface. In any case, hydrophobic interactions always promote adsorption, even if the associated energy is usually small. Moreover, they contribute to collective adsorption mechanisms since aggregative behaviors are observed near surfaces even when PFAS concentration falls below the critical micelle concentration (CMC) [47, 54, 55, 56, 57, 58, 59].

Some authors have identified adsorption parameters to quantify the contribution of electrostatic and hydrophobic interactions [60, 61]. For instance, the free energy associated with PFAS adsorption on ionic liquid‐modified clays shows that both contributions are involved in the adsorption process [60]. More generally, the correlation between adsorbed quantities and PFAS charge/dipole or hydrophobicity can be used to assess which interactions prevail [53, 62]. For hydrophobic interactions, two quantities can be considered: (1) the hydrophobicity indicator $\log D_{\mathrm{ow}}$ [63, 64] and (2) the half‐breakthrough bed volume $BV_{50}$ (a quantity referring to the volume of the fluid passing through an adsorbent bed until the output adsorbate concentration is equal to half of the input concentration and used as apparent adsorption capacity). A monotonic correlation between those two quantities indicates that an increase in PFAS hydrophobicity systematically leads to an increase in their retention [61]. In contrast, for PFAS adsorption on ion exchange resins, electrostatic interactions are the main process drivers as strong correlations were observed between the PFAS negative charge and the apparent equilibrium adsorbed amount $q_{e}$ [62]. Even when hydrophobic interactions are dominant, the impact of electrostatic interactions remains important, as can be concluded from studies on the effect of co‐ions and/or pH levels [65, 66].

#### 2.1.3 PFAS‐Specific Effects

While molecular interactions and resulting adsorption mechanisms pertain to any adsorbing compound on a surface, the specific chemistry of PFAS leads to additional phenomena. Such complex effects near the surface and within the solution in the presence of coexisting species contribute to PFAS adsorption (Figure 2b). Depending on several factors related to adsorbent, adsorbate and solution chemistry, a specific interaction may dominate under certain thermodynamic conditions. As a result, while studying PFAS adsorption in a simplified environment such as pure water allows identifying individual adsorption effects, it is also important to consider the interplay between multiple PFAS and other chemical compounds.

##### 2.1.3.1 Fluorine interactions

Molecular interactions with fluorine atoms in PFAS contribute to adsorption mechanisms. In particular, several authors have proposed that fluorophilic interactions occur between PFAS and fluorinated amine‐functionalized surfaces [67, 68, 69]. This specific interaction was invoked to explain enhanced PFAS adsorption and selectivity by fluorine‐containing materials. Other molecular interactions, such as Lewis acid‐base bonds involving fluorine atoms have also been reported [70]. However, these molecular interactions can be described as dipole–dipole or charge–dipole interactions, which allows classifying them into the categories listed above.

##### 2.1.3.2 Chemical adsorption

While out of the scope of this review, chemisorption is a possible adsorption mechanism. An important class of PFAS adsorbents consists of anion exchange resins (AER) based on adsorption through a chemical reaction. In more detail, an anion such as a surface hydroxyl group ${\mathrm{OH}}^{-}$ is exchanged with the aqueous solution and the charged adsorption site created by the removal of the hydroxyl group is then occupied by the anionic headgroup of the PFAS molecule [71]. A similar process occurs on metal–oxide surfaces containing hydroxyl groups [72].

##### 2.1.3.3 Organic matter

As discussed later, a fraction of the organic matter in water matrices shares similarities with PFAS (size, electrostatic charge, and hydrophobicity). Consequently, below certain concentration thresholds, OM may enhance PFAS adsorption through hydrophobic interactions, as an adsorbed organic compound serves as a hydrophobic binding site for a PFAS molecule (Figure 2b). Other collective effects include simultaneous adsorption of PFAS‐OM complexes [73].

##### 2.1.3.4 Co‐ions

Coexisting ions significantly impact adsorption as they affect charge – charge interactions. Various studies have demonstrated the existence of cationic bridges between charged entities [74, 75, 76, 77]. A cationic bridge between a negative surface and an anionic PFAS facilitates PFAS adsorption (Figure 2(b)), even under conditions where the adsorbent surface chemistry is not favorable for direct PFAS adsorption [74, 75, 76]. Cations may also promote PFAS aggregates formation by bridging anionic PFAS heads [77].

### 2.2 Static and Dynamic Adsorption

#### 2.2.1 Thermodynamics and Physical Models

Adsorption can be rationalized through physical models that capture both adsorption thermodynamics and kinetics. These models describe the adsorption isotherm (i.e. the number of adsorbed molecules per unit of surface area or mass of the adsorbing solid at equilibrium) and/or the adsorption kinetics (i.e. the time evolution of the adsorbed amount starting from given thermodynamic conditions). Let us first define the key variables and conventions. Unless stated otherwise, q(t) and c(t) denote the PFAS adsorbed amount at the surface and the PFAS bulk concentration within the solution at a given time t. These quantities are related through a mass balance equation:

(2) $$\begin{array}{c} q\left(t\right)-q\left(0\right)=\frac{V}{m_{s}}\left[c(0)-c(t)\right] \end{array}$$

where V is the solution volume and $m_{s}$ the solid adsorbent mass. In the above equation, c(0)−c(t) is the PFAS concentration difference in the solution between the times t=0 and t. Similarly, q(t)−q(0) is the PFAS adsorbed amount difference between the times t and t=0. The adsorption capacity $q_{\infty }$, which is obtained for an infinite concentration c→∞, corresponds to the maximum number of PFAS that can be adsorbed on the surface. We also define $q_{e}=q\left(\infty \right)$ and $c_{e}=c\left(\infty \right)$ as the adsorbed amount and solution concentration reached at equilibrium. With these notations, q(t) corresponds to the adsorbed amount per adsorbent mass (other notations are sometimes used, such as the adsorbed amount per surface area unit). In most adsorption experiments, the initial conditions are c(t)=q(t)=0 for t<0 and an initial concentration c(0)≠0 is injected at t=0. This is typically the procedure used in batch experiments that are repeated for many different c(0) to determine the entire adsorption isotherm $q_{e}\left(c_{e}\right)$ (with such conditions, Equation (2) should be used with q(0)=0). More generally, Equation (2) should be used, as it encompasses situations where q(0) at the injection time is non‐zero. This is the case in multi‐step adsorption experiments in which the adsorption isotherm is obtained by performing n concentration increments.

There is a large body of theoretical and empirical models to describe adsorption. Some models (e.g. Freundlich, Sips, Redlich–Peterson, Marczewski–Jaroniec [78]) rely on empirical approaches so their use is limited to providing a mathematical description of adsorption data. This can be very useful with the objective of efficiently fitting experimental data. Yet, because of the lack of physical foundations, these models should only be considered for fitting/description purposes – i.e. without considering the inferred parameters as robust and reliable physical quantities. Considering that the present review aims to understand PFAS adsorption on surfaces, we only focus on classical models that are based on a rigorous thermodynamic and/or kinetic description. In contrast, empirical models such as the Freundlich or Sips models are not discussed as they cannot be derived from theoretical considerations. In particular, despite their frequent and successful use in fitting experimental data, these models do not verify important theoretical limits (Henry regime at low concentrations, where $q_{e}\propto c_{e}$ and saturation where $q_{e}\to q_{\infty }$ at high concentrations) [79].

The *adsorption isotherm* is the most important quantity when considering adsorption equilibrium of a compound on a surface. It expresses the adsorbed amount $q_{e}$ as a function of the bulk concentration $c_{e}$ at equilibrium (i.e. for t→∞). Although adsorption and desorption constants provide key information on the affinity of the solid toward the adsorbate, they also contain relevant kinetic aspects.

##### 2.2.1.1 Henry model

The Henry model, which only applies to low concentrations c, is the simplest physical model for adsorption. Because the concentration $c_{e}$ is very low, the adsorbing solid surface does not saturate and $q_{e}$ is proportional to $c_{e}$:

(3) $$q_{e}\left(c_{e}\right)=k_{H}c_{e}$$

**FIGURE 3:**
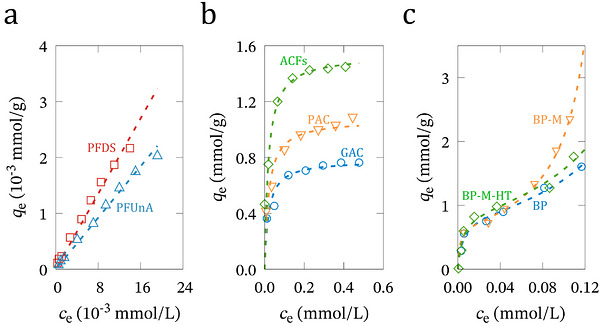
PFAS adsorption follows classical models such as (a) Henry model at low concentrations $c_{e}$, (b) Langmuir model or (c) BET model at high concentrations $c_{e}$. The symbols represent experimental data while the dotted lines are fits against the adsorption isotherm model. (a) Adsorption of PFDS and PFUnA on freshwater sediments made of silt (38%), sand (31%), and clay (31%) [65]. (b) PFOS adsorption on granulated (GAC), powdered (PAC), and fibers (ACFs) activated carbon exhibits a Langmuir‐like adsorption isotherm [80]. (c) PFOS adsorption on pure (BP), melamine‐functionalized (BP‐M), and heat‐treated melamine‐functionalized (BP‐M‐HT) carbon follows a BET adsorption isotherm model [54].

This equation corresponds to a mass balance condition between the PFAS adsorption and desorption constants at the surface. In particular, the Henry constant $k_{H}=k_{A}/k_{D}$ is defined as the ratio of the adsorption $k_{A}$ and desorption $k_{D}$ constants. At very low concentrations $c_{e}$, the adsorption of PFDS and PFUnA (length l=10) on freshwater sediments follows the Henry regime (Figure 3a) [65]. By deriving the Henry model (or Langmuir model below) from physical considerations, the adsorption constant $k_{H}=k_{A}/k_{D}$ is found to relate to the adsorption enthalpy ΔH i.e. $k_{H}\propto \exp\left(-\Delta H/k_{B}T\right)$. This fundamental adsorption quantity is connected to the adsorption potential energy U (described in Section 2.1), the pressure P, and the volume V of the adsorbed phase (H=U+PV). Therefore, comparing PFDS and PFUnA adsorption in Figure 3a, we conclude that the adsorption enthalpy at low concentrations is larger (more negative) for the sulfonate acid PFDS than for the carboxylate acid PFUnA. As explained in detail in Section 3, while these two PFAS share the same chain length, the adsorption of the sulfonate group is favored over the carboxylate group.

##### 2.2.1.2 Langmuir model

The most common physical model to describe adsorption is the Langmuir adsorption isotherm. In contrast to Henry model, which is limited to low bulk concentrations $c_{e}$, the Langmuir model accounts for surface saturation when the adsorbed amount $q_{e}$ becomes non‐negligible compared to the maximum adsorption capacity $q_{\infty }$ (for large $c_{e}$):

(4) $$q_{e}\left(c_{e}\right)=q_{\infty }\frac{k_{L}c_{e}}{1+k_{L}c_{e}}$$

where the Langmuir constant is defined from the adsorption and desorption constants, i.e. $k_{L}=k_{A}/k_{D}$. Here, all adsorption sites (1) can adsorb only one molecule and (2) are assumed to be independent (as a result of the first approximation, this model only predicts monolayer adsorption). The Langmuir model allows capturing the behavior of certain PFAS in simple water matrices, as illustrated for PFOS adsorption on active carbons (AC) in Figure 3b. Fitting experimental data to the Langmuir model provides fundamental insight into PFAS adsorption. For instance, the adsorption capacity $q_{\infty }$ is related to the specific surface area or the porous volume of the adsorbent. However, the Langmuir model is limited since it assumes no lateral interactions between adsorbed molecules and only allows monolayer adsorption.

##### 2.2.1.3 BET model

In some cases, non‐negligible departure from the Langmuir model and its extensions are observed in PFAS adsorption at large concentrations $c_{e}$ [55, 81, 82]. In such situations, the Brunauer–Emmett–Teller (BET) model is sometimes found to accurately describe the adsorption process. This model describes adsorption as a multilayer process in which each adsorbed molecule becomes a new site that can adsorb another adsorbate molecule (the adsorption energy for the first adsorbed molecule on the surface is $\varepsilon_{0}$ while the adsorption energy for any subsequently adsorbed molecule is ε). The BET adsorption isotherm for liquid phase adsorption writes [83]:

(5) $$q_{e}=\frac{q_{\infty }kc_{e}}{\left[1-k_{L}c_{e}\right]\left[1+\left(k-k_{L}\right)c_{e}\right]}$$

where $k_{L}$ is the Langmuir adsorption constant for the first adsorbed layer, while k is a constant characterizing multilayer adsorption beyond the first adsorbed layer and $q_{\infty }$ is the monolayer capacity (i.e. the number of molecules that can be adsorbed in a single layer). The different constants $k_{L}$, k and $q_{\infty }$ are usually determined by fitting the BET model to experimental data [84]. This model was successfully used to describe PFAS adsorption on carbon‐modified adsorbent (Figure 3c) [54].

As summarized in Table 1, alternative models were developed to capture more complex behaviors. The dual‐site Langmuir model accounts for the surface heterogeneity by introducing two adsorption site types (α, β), with different adsorption energies ($U^{\alpha }$, $U^{\beta }$) [85, 86]. For a multi‐adsorbate system containing n species, the multi‐component Langmuir model can be used [87]. However, this model is limited in its application to PFAS adsorption as it may not capture preferential interactions for short/long PFAS. Therefore, some authors have proposed a competitive model including a hydrophobicity parameter [88]. Many other physical models for multi‐solute adsorption have been developed and can be used for fitting multi‐component adsorption isotherms [78, 89]. Additionally, collective effects impact the adsorption of PFAS compounds, provided their concentration in solution reaches a certain threshold (depending on the affinity of the adsorbate molecule for the surface, this threshold can be far below or far above the CMC). In such cases, a model accounting for lateral interactions between adsorbed molecules is relevant [90, 91, 92, 93].

**TABLE 1:** Summary of the main physical models for the thermodynamics of surface adsorption. While other (semi‐)empirical models successfully fit experimental data (e.g. Freundlich, Sips), only models rigorously deriving from physical principles are reported here. The hypothesis behind the adsorption mechanism (surface saturation, lateral interactions (LI), mono‐ vs multi‐layers, homogeneous vs heterogeneous surface), and the shape of the adsorption isotherm (linear, L‐shape (with no inflection), S‐shape (with one inflection point) or LS‐shape (with two inflection points)) are provided.

Model	Adsorption mechanism	Isotherm shape
Henry	No surface saturation, no LI, monolayer, homogeneous surface	Linear
Langmuir [94]	Surface saturation, no LI, monolayer, homogeneous surface	L‐shape
Dual‐site Langmuir [85, 86]	Surface saturation, no LI, monolayer, heterogeneous surface	L‐shape
Multi‐component Langmuir	Surface saturation, no LI, monolayer, homogeneous surface	L‐shape
Competitive Multi‐component Langmuir [88]	Surface saturation, no LI, monolayer, homogeneous surface	L‐shape
BET [95]	Surface saturation, no LI, multilayer, homogeneous surface	S‐shape
Flory‐Huggins [96]	Surface saturation, LI (mean‐field), homogeneous surface	L‐ and S‐shape
Quasi‐Chemical Approx. [92]	Surface saturation, LI (local configurations, 2‐body, short‐range), monolayer, homogeneous surface	L‐ and S‐shape
Tempkins [91]	Surface saturation, LI (implicit), monolayer, homogeneous surface	L‐shape
Frumkin‐Fowler [91]	Surface saturation, LI (mean‐field, 2‐body, short‐range), monolayer, homogeneous surface	L‐ and S‐shape
Mean‐Field (I) [93]	Surface saturation, LI (mean‐field, 2‐,3‐body, short‐range), monolayer, homogeneous surface	L‐, S‐, and LS‐shape
Mean‐Field (II) [97]	Surface saturation, LI (mean‐field, 2‐body, short‐range), monolayer, heterogeneous surface	L‐ and S‐shape
Mean‐Field (III) [98]	Surface saturation, LI (mean‐field, 2‐body, long‐range), monolayer, homogeneous surface	L‐, S‐ and LS‐shape

#### 2.2.2 Kinetic Modeling

##### 2.2.2.1 Effective modeling

Adsorption equilibrium is essential to rationalize the behavior of PFAS in contact with surfaces, but it is often insufficient to understand the fate of the chemicals in practical situations as they involve kinetic aspects. In particular, elucidating the dynamical processes ruling PFAS adsorption is needed to determine how adsorption and transport couple at different scales – from the vanishing molecular level to the macroscopic engineering level [99]. The most general model to describe the time evolution of the adsorbed amount q(t) is a kinetic equation of order n:

(6) $$\begin{array}{cc} \frac{dq(t)}{dt} & =k{\left[q_{e}-q\left(t\right)\right]}^{n} \end{array}$$

where q(t) converges toward equilibrium $q_{e}$ at a rate depending on $q_{e}-q\left(t\right)$. The parameters k and n describe the reaction rate and the kinetic order. Although this general framework is used when an empirical description is sought [100], there is a limited set of n that corresponds to physical kinetics. While the reaction order n=0 is associated with a total reaction in which only the forward reaction occurs (without any counter‐desorption), the reaction order n=1 corresponds to first‐order kinetics which are used to model adsorption. In contrast to these physical situations, other reaction orders do not necessarily correspond to an underlying physical model. For instance, the reaction order n=2 is often employed for fitting purposes [101] even though purely second‐order kinetics do not derive from any physical descriptions. In fact, it has been suggested that such kinetics only correspond to the limit of large concentration variations [102] or to strongly heterogeneous systems [102, 103]. Nevertheless, first‐ and second‐order kinetics are frequently used in practice to assess whether adsorption pertains to physical or chemical phenomena [71, 76, 101, 104, 105], even though the general validity of second‐order kinetics has not been clearly established. Other effective models, including the intraparticle diffusion model and the pseudo‐n‐order kinetic model, have been employed for modeling PFAS adsorption kinetics [99, 106].

##### 2.2.2.2 Physical modeling

Adsorption kinetics correspond to a mass‐balance equation between adsorption and desorption of adsorbate A on a surface site S: A+S⇌AS. The adsorption and desorption rates $k_{A}$ and $k_{D}$ depend on physical parameters limiting or favoring adsorption: (1) the surface adsorption capacity determined by the number of accessible sites, (2) the adsorbate bulk concentration, and (c) the adsorption energy.

**FIGURE 4:**
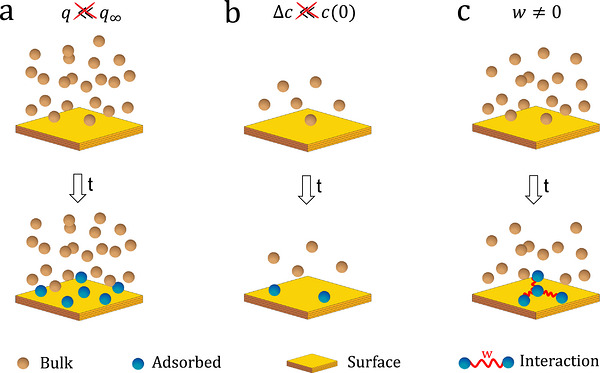
Adsorption kinetics can be described with different models according to the complexity of the system. The models presented here show departure from the conventional picture in which the surface saturation, reservoir depletion, and lateral interactions are neglected. (a) When the surface has a limited adsorption capacity $q_{\infty }$, the adsorption process is limited by the number of available sites $q_{\infty }-q\left(t\right)$. (b) When the reservoir is small, the variation in bulk concentration Δc is significant upon adsorption. Classical kinetic models should be adapted accordingly to account for the departure of the instantaneous bulk concentration c(t) from its initial value c(0) [93]. (c) For complex compounds such as PFAS, the lateral interaction energy w is non‐negligible. In particular, when the affinity toward the surface is low, their adsorption can be driven by cooperative effects rather than by single‐body interactions between the surface and a single PFAS [93].

Adsorption is necessarily constrained by a saturation term corresponding to the maximum adsorption capacity $q_{\infty }$. As the number of adsorbed molecules increases, the number of adsorbing sites reduces concomitantly (Figure 4a). As a result, the adsorption capacity left at time t is given by $q_{\infty }-q\left(t\right)$. Adsorption is also affected by depletion effects within the reservoir containing PFAS in solution with a concentration c(t) (Figure 4b). These effects occur as the decrease in c(t) becomes non‐negligible with respect to the initial concentration c(0). Invoking the rescaled bulk concentration $\tilde{c}\left(t\right)=c\left(t\right)V/m_{s}$, such effects occur whenever $\tilde{c}\left(0\right)-\tilde{c}\left(t\right)$ becomes comparable to q(t) [here we use the rescaled concentration $\tilde{c}$ as it allows direct comparison with q]. Another important aspect is the definition of the adsorption and desorption constants $k_{A}$ and $k_{D}$ which, as we recall from the discussion on Henry isotherm, are linked to the enthalpy of adsorption. In simple cases,, $k_{A}$ and $k_{D}$ are constant. However, in more complex media, they depend on pH and on the presence of co‐existing species (e.g. ions, organic matter). Moreover, when adsorbed species undergo lateral interactions (Figure 4c), $k_{A}$ and $k_{D}$ can even depend on the adsorbed amount q [90, 93].

**FIGURE 5:**
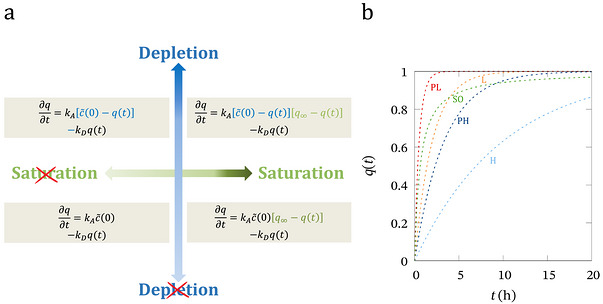
(a) The kinetic equations corresponding to each model are summarized in this diagram. When saturation and depletion are neglected (bottom left equation), the kinetics are described by the first‐order Henry model. Moving to the right part of the diagram, saturation is taken into account and leads to the Langmuir kinetics. When depletion is included while saturation is neglected (top left), the kinetics are given by the pseudo–Henry model. Finally, when both saturation and depletion are considered (top right), the kinetics follow the pseudo–Langmuir model. (b) The corresponding (normalized) analytical solutions are depicted in the figure. For an infinite reservoir, and an unlimited adsorption capacity, the adsorption kinetics follow a first‐order (FO) Henry (H) model. When saturation is taken into account, the FO kinetics follow a Langmuir (L) model. Including only depletion and neglecting saturation leads to pseudo–Henry (PH) kinetics. When both surface saturation and reservoir depletion occur, the kinetics follow a mixed order (FO + SO) pseudo–Langmuir (PL) model. Finally, the literature also reports in specific cases a second‐order (SO) kinetic model.

At a given time t, the adsorption kinetics can be described by the instantaneous adsorption rate $k_{A}\tilde{c}\left(t\right)\left[q_{\infty }-q\left(t\right)\right]$ and desorption rate $k_{D}q\left(t\right)$. If the instantaneous rescaled bulk concentration $\tilde{c}\left(t\right)$ is homogeneous (which applies for a well‐agitated reservoir, or in the limit of infinitely fast diffusing systems), the instantaneous adsorption term can be replaced by $k_{A}\left[\tilde{c}\left(0\right)-q\left(t\right)\right]\left[q_{\infty }-q\left(t\right)\right]$. Using a simple mass balance condition, these terms lead to the following constitutive equation:

(7) $$\frac{dq(t)}{dt}=k_{A}\left[\tilde{c}\left(0\right)-q\left(t\right)\right]\left[q_{\infty }-q\left(t\right)\right]-k_{D}q\left(t\right)$$

where we recall that $\tilde{c}\left(t\right)$ = $c\left(t\right)V/m_{s}$. Taking into account both depletion and saturation effects, the adsorption kinetics in Equation (7) becomes a mixed‐order model containing first‐order (n=1) and second‐order (n=2) kinetics. Although this model was explored in some theoretical approaches [107, 108], it was not applied to PFAS adsorption. To solve the complex kinetics described in Equation (7), we invoke the roots of the second‐order polynomial in q appearing on the right‐hand side of Equation (7): $\xi_{\pm }=\left[\left(k_{A}q_{\infty }+k_{A}\tilde{c}\left(0\right)+k_{D}\right)\pm \sqrt{\Delta }\right]/2k_{A}$ with $\Delta ={\left(k_{A}q_{\infty }+k_{A}\tilde{c}\left(0\right)+k_{D}\right)}^{2}-4k_{A}^{2}q_{\infty }\tilde{c}\left(0\right)\geq 0$. This allows recasting Equation (7) and obtaining its solution:

(8) $$\begin{array}{c} \begin{array}{cc} \frac{dq(t)}{dt} & =k_{1}\left[q_{e}-q\left(t\right)\right]+k_{2}{\left[q_{e}-q\left(t\right)\right]}^{2}\\ q(t) & =\frac{\xi_{+}-\xi_{+}e^{\sqrt{\Delta }t}}{1-\frac{\xi_{+}}{\xi_{-}}e^{\sqrt{\Delta }t}} \end{array} \end{array}$$

where $q_{e}=\xi_{-}$ is the equilibrium adsorbed quantity, $k_{1}=k_{A}\left(\xi_{+}-\xi_{-}\right)=\sqrt{\Delta }$ is the first‐order kinetic coefficient and $k_{2}=k_{A}$ is the second‐order kinetic coefficient. The analytical expression q(t) is illustrated with the red curve in Figure 5b. As described in what follows, some simplifications allow retrieving classical kinetic models such as Henry or Langmuir kinetics.

##### 2.2.2.3 •Henry model (first‐order kinetics without saturation/without depletion)

When surface saturation, reservoir depletion, and all lateral interaction effects can be neglected, the general kinetic model described in Equation (7) simplifies to first‐order kinetics corresponding to Henry's regime [101, 108, 109]:

(9) $$\begin{array}{c} \begin{array}{cc} \frac{dq(t)}{dt} & =k_{A}\tilde{c}\left(0\right)-k_{D}q\left(t\right)\\ q(t) & =k_{H}\tilde{c}\left(0\right)\left[1-\exp\left(-k_{H}t\right)\right] \end{array} \end{array}$$

The solution of this kinetic model q(t) corresponds to the light blue curve in Figure 5b. In the long‐time limit, the equilibrium value falls on the curve associated to Henry adsorption isotherm [Equation (3)].

##### 2.2.2.4 •Langmuir model (first‐order kinetics with saturation/without depletion)

In some cases, the adsorbed amount q(t) is not negligible with respect to the maximum adsorption capacity $q_{\infty }$ (Figure 4a). In such situations, provided that reservoir depletion and lateral interactions can be neglected, the general kinetic model described in Equation (7) leads to first‐order kinetics corresponding to the Langmuir model:

(10) $$\begin{array}{c} \begin{array}{cc} \frac{\mathrm{dq}(t)}{\mathrm{dt}} & =k_{A}\tilde{c}\left(0\right)\left[q_{\infty }-q\left(t\right)\right]-k_{D}q\left(t\right)\\ q(t) & =\frac{k_{L}\tilde{c}\left(0\right)q_{\infty }}{1+k_{L}\tilde{c}\left(0\right)}\left[1-\exp(\left[k_{A}\tilde{c}\left(0\right)+k_{D}\right]t)\right] \end{array} \end{array}$$

The solution q(t) is plotted in orange in Figure 5b. Similarly, the adsorption kinetics associated with the dual‐site Langmuir model is [110]:

(11) $$\begin{array}{c} \frac{dq(t)}{dt}=\frac{dq_{\alpha }\left(t\right)}{dt}+\frac{dq_{\beta }\left(t\right)}{dt}=x_{\alpha }\left[\tilde{c}\left(0\right)k_{A}^{\alpha }\left[q_{\infty }-q\left(t\right)\right]-k_{D}^{\alpha }q\left(t\right)\right]\\ +x_{\beta }\left[\tilde{c}\left(0\right)k_{A}^{\beta }\left[q_{\infty }-q\left(t\right)\right]-k_{D}^{\beta }q\left(t\right)\right] \end{array}$$

where $x_{\alpha }$ and $x_{\beta }=1-x_{\alpha }$ are the fractions of sites of type α and β. Each site type is also associated with an adsorption energy $U^{\alpha ,\beta }$ yielding different adsorption and desorption constants $k_{A}^{\alpha ,\beta }$ and $k_{D}^{\alpha ,\beta }$.

##### 2.2.2.5 •Pseudo‐Henry model (first‐order kinetics with depletion/without saturation)

When adsorption is only limited by the finite size of the solution, the variation in the bulk concentration Δc(t)=c(0)−c(t) must be included (Figure 4b) and the general kinetic model in Equation (7) becomes:

(12) $$\begin{array}{c} \begin{array}{cc} \frac{dq(t)}{dt} & =k_{A}\left[\tilde{c}\left(0\right)-q\left(t\right)\right]-k_{D}q\left(t\right)\\ q(t) & =\frac{k_{A}\tilde{c}\left(0\right)}{k_{A}+k_{D}}\left[1-\exp\left([k_{A}+k_{D}]t\right)\right] \end{array} \end{array}$$

The solution q(t) for this model is the dark blue curve in Figure 5b. While most kinetic models are based on the implicit assumption that the bulk concentration is constant, i.e. $c\left(t\right)=c\left(0\right)=c_{e}$ at all times t (infinite reservoir/solution), this assumption is correct only for Δc(t)<<c(0). However, in many practical situations, this approximation breaks down, and adsorption is affected by depletion. In fact, in batch experiments, the adsorbed amount q(t) is usually estimated by measuring the difference between the initial and final bulk concentrations: q(t)−q(0)∼c(0)−c(t). If variations in the bulk concentration are negligible (as assumed in the Langmuir model), q(t) cannot be measured with this method. Consequently, experimental data should be analyzed carefully in practice as the underlying kinetics can involve non‐conventional Langmuir or Henry models.

##### 2.2.2.6 •Lateral interactions and cooperativity

Cooperative effects are expected for complex compounds such as PFAS as their adsorption can involve lateral interactions. In such cases, adsorption kinetics can be described with a first‐order cooperative model for the adsorption of aggregated monomers [90]. Alternatively, a kinetic model including all factors influencing PFAS adsorption (i.e. surface saturation, reservoir depletion and lateral interactions) has recently been developed [93]. In this approach, a lattice gas model accounting for many‐body interactions is solved using a mean‐field approximation, yielding a thermodynamic and kinetic framework for the complex adsorption processes that PFAS may undergo.

## 3 Specificities of PFAS Adsorption in Practical Conditions

### 3.1 Impact of PFAS and Surface Properties

#### 3.1.1 PFAS Functional Group, Dissociation, Chain Length, and Phase Diagram

##### 3.1.1.1 Functional groups and dissociation

The impact of PFAS headgroup depends on its charge in solution as it dictates the resulting electrostatic interactions. Most common PFAS compounds (i.e. per‐ and polyfluoroalkyl acids (PFAAs)) are often assumed to be anionic under general practical conditions but this assumption is not always appropriate [111, 112, 113]. In particular, PFAAs dissociated state is governed by their acid dissociation constant $pK_{a}$, which determines the dissociation degree at a given pH. Thus, the $pK_{a}$ plays a major role as it leads to different adsorption behavior for PFAS presenting similar hydrophobicity and similar net charge once dissociated [46, 114, 115]. Usual pH ranging from 6.5 to 8.5 surpass $pK_{a}$ for most PFAS, therefore resulting in predominantly anionic compounds. However, certain PFAS with high $pK_{a}$ may maintain a non‐negligible fraction of neutral molecules at average pH. Consequently, depending on their $pK_{a}$, the fraction of dissociated PFAS at a given pH varies. Upon increasing pH, neutral PFAS dissociate further and the fraction of anionic forms increases. This specificity explains the important differences among PFAS with identical chain lengths and net charges when dissociated in water. For instance, FOSA and PFOS possess the same chain length l=8 and charge when dissociated but they do not have the same functional group = ${\mathrm{SO}}_{3}^{-}$ for PFOS ($pK_{a}$ = –3.2) and ${\mathrm{SO}}_{2}{\mathrm{NH}}_{2}$ for FOSA ($pK_{a}∼6$). Consequently, most PFOS molecules (as well as any other super acid characterized by an extremely low $pK_{a}$ value), are in their dissociated state for any pH, while most FOSA molecules remain neutral over a significant range of pH [116].

**FIGURE 6:**
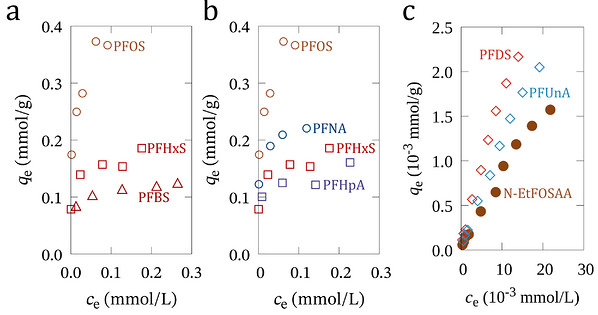
Adsorption of different PFAS on (a,b) ionic liquid‐modified clay [60] [dominant electrostatic interactions] and (c) sediments [65] (dominant hydrophobic interactions). In these panels, sulfonic acids (PFSAs) are represented in brown while carboxylic acids (PFCAs) are represented in blue. PFAS with the same chain length l are represented with the same symbol: triangles (l=4), squares (l=6), circles (l=8) and diamonds (l=10). PFAS in their neutral state are shown using filled symbols while PFAS in their dissociated state are shown using empty symbols. In panel (a), adsorption increases with l as observed from the adsorption of PFBS (l=4), PFHxS (l=6) and PFOS (l=8). (b) The role of the functional headgroup is studied by comparing the adsorption of PFAS with l=8 (PFOS and PFNA) and l=6 (PFHxS and PFHpA). For a fixed fluoroalkyl chain length l, the dissociated sulfonic PFAS (brown) adsorbs more than the dissociated carboxylic PFAS (blue). (c) Effect of the headgroup and chain length l upon adsorption driven by hydrophobic interactions: PFDS and PFUnA both possess a chain length l=10 but the dissociated sulfonic PFAS is more strongly adsorbed under the same concentrations in water. N‐EtFOSAA is less adsorbed than the other two compounds as N‐EtFOSAA molecules are shorter (l=8) and mostly neutral under the experimental conditions ($pK_{a}$ ≥ pH).

Even when two PFAS are in the same anionic state, their adsorption can differ significantly because the exact charge distribution on their headgroup leads to different electrostatic‐driven interactions. For instance, carboxylate (PFCAs) and sulfonate (PFSAs) PFAS are often compared because they exhibit different physicochemical properties even at very high pH, where most acidic PFAS are anionic. Figure 6b,c shows that PFSAs are more adsorbed and less impacted by competition with other PFAS than PFCAs – despite their similar chain length l and net charge [60]. These differences result from the polarity difference between the functional headgroups. Even if ${\mathrm{SO}}_{3}^{-}$ and ${\mathrm{COO}}^{-}$ headgroups have the same net charge, the sulfonate group presents a more delocalized charge and is more polarizable [117, 118]. Additionally, for the same chain length l, PFSAs are more hydrophobic than PFCAs [70, 77]), therefore leading to increased lateral interactions and stronger repulsion from water for PFSAs.

##### 3.1.1.2 Chain length

When evaluating PFAS adsorption based on their length l, the number of ${\text{CF}}_{2}$– or ${\text{CF}}_{3}$– moieties rather than the number of carbon atoms must be considered. Indeed, it is the moiety and not the carbon atom alone that contributes to the hydrophobicity of the molecule (every ${\text{CF}}_{2}$– moiety contributes by 0.50–0.60 log units to the distribution coefficients $D_{\mathrm{ow}}$ [65]). In particular, when comparing PFOS and PFOA, it is frequently assumed that their chain length l is the same [46, 119, 120]. Yet, even though both molecules contain 8 carbons, PFOS and PFOA differ in the number of ${\text{CF}}_{2}$– and, hence, in their chain length l. A more reliable comparison should thus involve PFOS and PFNA which differ only in their functional group (sulfonate vs carboxylate).

Several studies have demonstrated preferential adsorption for longer PFAS (Figure 6a,b) [60, 115, 121, 122]. This effect is attributed to the increase in PFAS hydrophobicity upon increasing l [61, 63], resulting in lower solubility and enhanced hydrophobic interactions with hydrophobic surfaces [123]. While it is significant for adsorption on hydrophobic surfaces, this effect also impacts electrostatically‐driven adsorption as it increases the repulsion of water toward PFAS. Additionally, the critical micelle concentration being strongly connected to the PFAS chain length l [63], longer PFAS exhibit enhanced attractive lateral interactions which potentially lead to cooperative behaviors. Despite its positive impact on PFAS affinity toward the surface, a longer chain l impacts the diffusion of a PFAS molecule. In fact, the equilibration time increases systematically with l [57, 76]. Moreover, in microporous adsorbents, longer PFAS chains restrict access to a large number of adsorption sites [77] and lead to pore blockage. In that case, surface morphology is a key factor that influences the adsorption kinetics of long PFAS compounds.

##### 3.1.1.3 Phase Diagram

Owing to their amphiphilic nature, PFAS share the same physical chemistry behavior as other surfactant molecules. In particular, their solubility (and thus their affinity toward solid surfaces) is strongly dependent on temperature/concentration, with a rich behavior in aqueous solutions. An important feature of surfactants is their Krafft point $T_{K}$ [124], which is defined as the triple point where the three following states coexist: (1) monomers solubilized in water, (2) micelles above the critical micelle concentration (CMC) and (3) liquid crystal phase. In other words, this temperature corresponds to the intersect of the monomer solubility curve s(T), the CMC temperature curve CMC(T), and the phase transition line of hydrated monomers to micelles and/or liquid crystals. For $T<T_{K}$, the PFAS solubility is very low so that PFAS undergo a transition from the hydrated monomer state to the liquid crystal state upon increasing their concentration. In contrast, for $T>T_{K}$, the PFAS solubility is not low due to the significant entropy contribution at high temperature so that PFAS undergo a transition from the hydrated monomer state to the micellar state. In the latter case, it is possible to identify the CMC, i.e. the concentration threshold at which surfactants self‐aggregate into micelles within the solvent [124]. Notably, surface aggregates may form at concentrations lower than the CMC [47]. Thus, even at concentrations 10 to 100 times below the CMC, PFAS adsorption on solid surfaces may be strongly impacted by cooperative behaviors, which should motivate the use of models including lateral interactions when fitting adsorption data. More generally, at low temperatures, PFAS compounds form ordered structures which do not mix well with water. Miscibility is only observed for low concentrations and is favored by an increase in the temperature T – as high temperatures increase the probability of observing a disordered state (i.e. solvated monomers) over an ordered state (i.e. liquid crystal or micelle).

#### 3.1.2 Surface Geometry and Chemistry

##### 3.1.2.1 Particle/pore size

Studies on activated carbons (AC) have shown that adsorption on powdered AC (PAC) is faster than on granular AC (GAC) [125]. Some authors attribute this observation to the reduced particle size and corresponding larger external surface area in PAC. More generally, adsorption is faster and more pronounced on adsorbents with smaller particle sizes [58, 126, 127]. The impact of pore size and interlayer structure was also studied to better understand their role in PFAS adsorption efficiency [76, 128]. Both small and large pores lead to beneficial and detrimental adsorption effects [70, 123]. On the one hand, adsorbents showing a large fraction of small pores (nanopores) present enhanced selectivity for small PFAS. Such adsorbents are also more selective in the presence of large‐size natural OM, therefore reducing potential competition with non‐targeted OM. However, small pore size also leads to slower adsorption kinetics because of pore blockage. On the other hand, larger pores are much more accessible – especially for longer PFAS [123]. Moreover, the combined presence of mesopores (2 – 50 nm) and specific surface chemistry allows multilayer PFAS adsorption – an adsorption regime formally described using the BET model [54]. Larger pores can also induce competitive adsorption; in addition to reduced selectivity toward PFAS over OM, small PFAS adsorption is no longer favored with respect to longer PFAS (which generally overcome competitive adsorption due to stronger hydrophobicity). With such considerations, owing to their tunable pore size and high surface area, metal–organic‐frameworks (MOFs) appear as promising adsorbents for selective PFAS removal (with specific pore size and surface area that are selected based on the targeted PFAS [129, 130]). Similarly, covalent‐organic‐frameworks (COFs) offer tunable chemical structures and porosity, as well as high specific surface areas. However, as highlighted in Section 4.2, the practical implementation of these novel technologies still requires further investigation, as current synthesis methods remain costly while the long‐term stability and possible regeneration of these new adsorbents have not been fully characterized yet [131, 132].

##### 3.1.2.2 Surface affinity

Measuring the surface area to quantify the number of adsorption sites is often insufficient to predict the adsorption capacity of an adsorbent. The chemical nature of the surface also plays a role, as it governs the type and strength of molecular interactions with PFAS. When electrostatic interactions dominate PFAS adsorption, the surface affinity and the adsorbed amount can be characterized through the zeta‐potential. The latter is influenced by the pH level: as pH increases, the zeta‐potential decreases making electrostatic interactions between negatively charged PFAS and the surface less attractive [57]. Consequently, PFAS partitioning at the surface decreases following a similar trend [133]. However, while the zeta‐potential is an important descriptor, affinity toward negatively charged PFAS also depends on other surface properties, such as the anion (AEC) and cation (CEC) exchange capacities. For “naturally” protonated adsorbents, AEC is a good indicator of the adsorption site density, and it can be compared to the maximal adsorbed amount of anionic PFAS [134]. Although a good correlation is typically observed for homogeneous surfaces, strong surface heterogeneity makes some sites inaccessible, thereby weakening the correlation between AEC and maximal adsorbed amount. Furthermore, when an initially negatively charged surface becomes positively charged upon cation binding, CEC provides a more reliable estimate of the number of available cationic sites than the zeta‐potential [60]. Even when they are not the dominant mechanism, hydrophobic interactions [53, 57, 135, 136], hydrogen bonds, Lewis acid‐base interactions [70], ligand exchange and fluorophilic interactions (achieved through the addition of fluoroalkyl groups at the surface) [67, 126, 137] can also be exploited to design efficient adsorption‐based PFAS remediation techniques through surface functionalization. In this context, some authors have proposed linkage functionalization of three‐dimensional frameworks, such as covalent‐organic frameworks (COFs) with amine groups. In addition to a faster functionalization process, the authors have shown that the newly designed adsorbent is significantly more efficient at adsorbing PFOA than previous COF designs [138].

### 3.2 Chemical Environment and Surrounding Conditions

#### 3.2.1 Impact of pH

**FIGURE 7:**
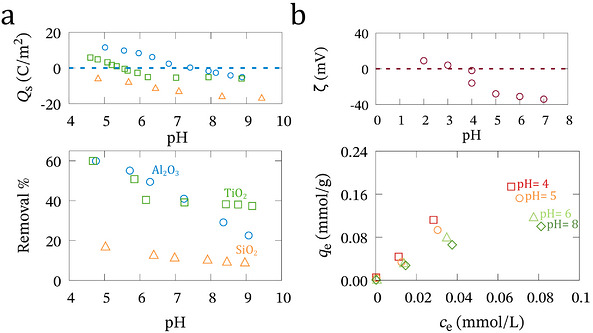
(a) Adsorption of PFOS on ${\mathrm{Al}}_{2}O_{3}$, ${\mathrm{TiO}}_{2}$, and ${\mathrm{SiO}}_{2}$ [139]. At low pH, most oxides (e.g. ${\mathrm{Al}}_{2}O_{3}$ and ${\mathrm{TiO}}_{2}$) are positively charged. When increasing the pH, the surface charge density ($Q_{s}$) decreases (top panel) as the surface gets deprotonated. As a result, the PFOS removal percentage decreases upon increasing the pH (bottom figure). When the surface is already negative over most of the pH range (e.g. ${\mathrm{SiO}}_{2}$), changing the solution pH does not strongly modify adsorption and, hence, PFOS removal percentage. (b) Adsorption of PFOS on negatively charged white graphene (i.e. hexagonal Boron Nitride) for pH = 4, 5, 6, and 8 [21]. The role of electrostatic interactions is highlighted through strong dependence on pH. Although the surface is negative for pH > 3.5 (see zeta‐potential for white graphene in the top panel), varying the pH from 4 to 8 impacts the PFAS adsorption isotherm (bottom panel).

Varying pH modifies both the adsorption capacity [57, 121] and the surface charge [75]. Additionally, depending on their acid dissociation constant ($pK_{a}$), varying the pH may also modify PFAAs dissociation state. While pH level plays a major role in mechanisms driven by electrostatic interactions, it also influences adsorption mechanisms dominated by hydrophobic interactions, as it enhances or reduces electrostatic repulsion between charged PFAS or between a negatively charged surface and an anionic PFAS [21, 57, 58, 77]. Typically, when adsorption is driven by electrostatic interactions, a pH increase induces surface deprotonation and a larger fraction of PFAAs (if not all) becomes anionic [133, 139]. As a result, the number of binding sites and the affinity of the surface toward anionic PFAS are both reduced (Figure 7a). Adsorption processes that are driven by non‐electrostatic interactions are also diminished upon increasing solution pH, as the repulsion between a negatively charged adsorbent and an anionic PFAS increases (Figure 7b) [21, 58]. However, in presence of Lewis‐acid sites on the adsorbent, the opposite effect can be observed [70]. Indeed, an increase in ${\mathrm{OH}}^{-}$ concentration upon increasing pH can facilitate the binding of basic fluorine atoms from the PFAS tail to the acidic sites of the surface. Another study conducted on the impact of pH and co‐ions concentrations suggests that in the presence of divalent cations, surface deprotonation allows the formation of cation bridges, therefore creating additional binding sites and enhancing PFAS adsorption [74].

#### 3.2.2 Salinity and Ionic Strength

**FIGURE 8:**
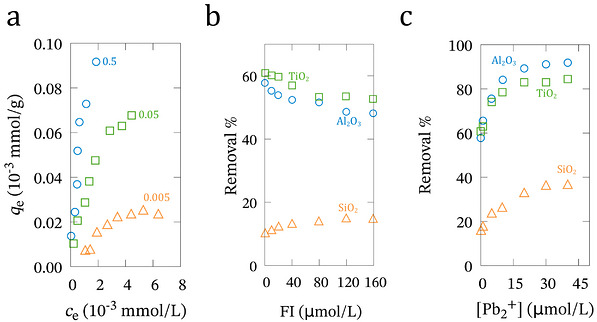
Impact of coexisting ions, ionic strength and cation bridging on PFOS adsorption on (a) sediments [74] and (b,c) nano‐oxides [139]. (a) PFOS adsorption on sediments increases upon increasing the concentration of ${\mathrm{CaCl}}_{2}$, going from 0.005, to 0.05 and to 0.5 $\mathrm{mol}.L^{-1}$ in a solution with neutral pH. These data can be rationalized by invoking the corresponding variation of the zeta potential from –10.3 to 8.35 mV. (b) When the surface is positively charged (which is the case for ${\mathrm{Al}}_{2}O_{3}$ and ${\mathrm{TiO}}_{2}$ at pH = 5), the ionic strength – which is controlled by the concentration of ${\mathrm{NaNO}}_{3}$ – inhibits PFOS adsorption. The opposite effect is observed on the negatively charged surface of ${\mathrm{SiO}}_{2}$. (c) Divalent cations such as ${\mathrm{Pb}}^{2+}$ enhance PFOS removal through cation bridging.

The presence of coexisting ions within the solution also plays a major role in PFAS adsorption through the concept of ionic strength. The latter is a function of the concentration $c_{i}$ of ions of type i:

(13) $$\begin{array}{c} I=\frac{1}{2}\sum_{i}c_{i}z_{i}^{2} \end{array}$$

where $z_{i}$ is the electrostatic charge carried by the ion species i. This expression enters in the definition of the Debye length, characterizing electrostatic screening within an electrolyte solution [140]. Experimentally, I is often evaluated by measuring other physical quantities [141]. For instance, using the electrolyte conductivity EC and the total solute concentration $C_{T}$, the ionic strength was estimated as $I=\frac{0.025\times C_{T}+0.083\times EC\times C_{T}}{C_{T}+0.121\times EC}$. In practice, increasing the salinity and hence the ionic strength I, induces a salting‐out effect of PFAS. In more detail, the solubility of these chemicals decreases with increasing I as electrostatic screening enhances the impact of hydrophobicity (i.e. repulsion from water molecules) [76, 142, 143, 144]. PFAS adsorption on kaolinite illustrates this effect, as increasing the concentration of monovalent ions enhances PFAS adsorption [145]. Additionally, higher cations concentration can increase the zeta‐potential of an initially negatively charged surface (Figure 8a) [74]. In such scenarios, PFAS adsorption is enhanced by the combination of the salting‐out effect and the beneficial change in surface chemistry. However, the salting‐out effect resulting from an increased cation concentration can be compensated by the electrical double layer compression [58, 77, 128]. In particular, for positively charged surfaces, an increase in the ion concentration compresses the electrical double layer which, in turn, diminishes the affinity of the surface toward anionic PFAS (Figure 8b). The balance between the salting‐out effect (enhancing PFAS repulsion from water) and the compression of the double electrical layer (reducing PFAS affinity) can explain the relatively minor impact of monovalent cations on acidic PFAS adsorption [65, 74, 77].

Increasing the concentration of divalent cations at constant I enhances anionic PFAS adsorption (Figure 8c). This effect can be rationalized by invoking cation bridging, [75, 144, 146, 147] which involves the formation of a cation bridge between anionic PFAS headgroups, reducing their electrostatic repulsion [77, 139] and favoring hydrophobic interactions between their tails, thus boosting surface aggregates formation. Divalent cations also possess the ability to bind to negative sites on the surface, therefore creating new cationic sites for the adsorption of negatively charged PFAS [77]. In contrast, while monovalent anions do not appear to compete with anionic PFAS [145], divalent anions can bind to positively charged surfaces and compete with negatively charged and short PFAS molecules [60, 67, 114, 148].

### 3.3 Coadsorption and Competitive Adsorption

#### 3.3.1 Organic Matter

From a molecular standpoint, and based on the specific interactions cited in Section 2.1.3, PFAS adsorption in waste and natural water is influenced by the water solution chemistry – whose composition often includes coexisting species. Among them, organic matter (OM) plays an important role as it shares similarities with PFAS while being present in much higher concentrations (the typical OM concentration is mg/L which largely surpasses the typical ng/L PFAS concentration) [149]. OM encompasses natural organic matter (NOM) and effluent organic matter (EfOM) [150]. The first includes all organic compounds that occur naturally in drinking water, while EfOM includes organic compounds found in waste water and primarily emitted by human activities. When investigating the effect of OM on PFAS adsorption, pure water samples containing a controlled concentration of representative OM (e.g. humic acids (HA) and fulvic acids (FA)) are used. While this experimental approach consists of a targeted study of the influence of OM on adsorption, working with more realistic water samples is also needed as other organic compounds (e.g. proteins, acids, etc.) lead to complex adsorption mechanisms.

**FIGURE 9:**
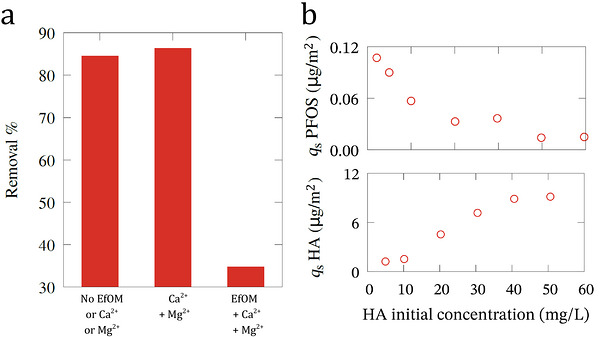
(a) PFOS adsorption on AC [152] is enhanced by the presence of divalent cations (${\mathrm{Ca}}^{2+}$ and ${\mathrm{Mg}}^{2+}$) while it is strongly reduced in the presence of organic matter (EfOM). (b) The negative impact of organic matter on PFOS adsorption is illustrated here by considering the amount of PFOS [top panel] and humic acid (HA) [bottom panel] that are adsorbed on boemithe ($q_{s}$ is the adsorbed amount per unit area of adsorbing surface) [155]. Upon increasing HA initial concentration, the amount of adsorbed PFOS decreases while the adsorbed amount of HA increases. This figure, which is consistent with the data shown in (a), illustrates the competition of PFOS/HA adsorption for the same adsorption sites.

The impact of OM depends on various factors, including their chemistry, size, concentration and surface characteristics. A non‐exhaustive list of possible effects of OM on PFAS adsorption includes: (1) competition between anionic PFAS and anionic OM for cationic sites (Figure 9) – especially when organic compounds and PFAS are of similar size [60, 76, 151, 152], (2) formation of HA‐PFAS complexes (Figure 2b), which can further co‐adsorb [73, 153] or induce pore blockage, (3) pore blockage induced by large OM [128, 154], and (4) reduction of electrostatic interactions between a positively charged surface and an anionic PFAS molecule due to preloaded anionic OM on the surface [133, 155, 156].

#### 3.3.2 Competitive Adsorption

**FIGURE 10:**
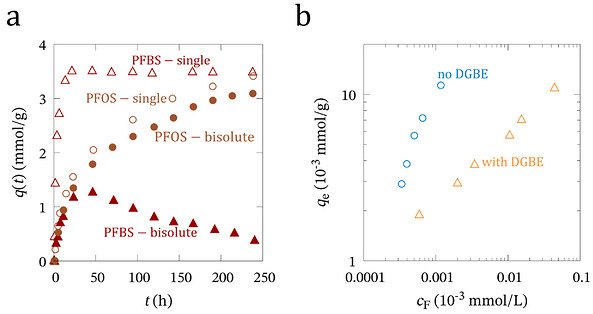
(a) Competitive adsorption on AER between PFOS (l=8) and PFBS (l=4) [159]. In single‐solute systems (empty symbols), both PFOS and PFBS reach a higher equilibrium adsorption capacity and equilibrium is reached faster in a single component system than in a two‐solute system. In the two‐solute solution (filled symbols), PFOS is dominating adsorption (its equilibrium value is barely affected by competition). Moreover, the adsorbed amount of PFBS decreases at t ∼ 50 h, showing more favorable adsorption for PFOS. (b) PFOS adsorption on colloidal AC is reduced in the presence of Diethylene Glycol Butyl Ether (DGBE) [157]. The adsorbed amount $q_{e}$ is shown as a function of the adsorption capacity $c_{F}$ extracted from a fit with the Freundlich model.

In most practical situations, PFAS adsorption involves natural surfaces and complex aqueous media. In these environments, PFAS invariably interact with co‐pollutants such as other PFAS with different physicochemical properties, heavy metals, and organic pollutants [139, 157, 158]. While the role of coexisting ions and organic matter was discussed above, we now consider competitive adsorption in multi‐solute systems. Within a single PFAS family (say with the same functional headgroup), long PFAS tend to favorably adsorb compared to shorter PFAS [53, 115, 126]. This favored adsorption arises from the higher hydrophobicity of long PFAS, whose solubility is lower and whose affinity toward hydrophobic adsorbents is stronger. In this context, stronger hydrophobic interactions often result in the replacement of already adsorbed short molecules by longer ones. Upon kinetic adsorption experiments, short PFAS molecules may diffuse faster toward the surface due to their smaller length l. Yet, after this transient time, longer PFAS replace shorter ones that were initially adsorbed. This behavior is illustrated in Figure 10a, which shows the adsorption kinetics of PFBS (l=4) and PFOS (l=8). After an initial increase in the adsorbed amount of PFBS (short chain), a subsequent decrease is observed as it is replaced by PFOS (long chain) [159]. Furthermore, for hydrophobic adsorbents in contact with a two‐solute system composed of PFOA (l=7) and GenX (l=4), the amount of adsorbed PFOA was higher than that of GenX when adsorbed in a single‐solute system [160]. This result indicates that in addition to competition between PFOA and GenX for the same adsorption sites, PFOA molecules can occupy additional sites that are not accessed by GenX molecules.

Beyond their chain length, the headgroup must also be considered in PFAS co‐adsorption. Indeed, while the removal capacity generally follows the hydrophobicity level for PFAS sharing the same headgroup, it may not be the case for solutions containing PFAS with different groups. In fact, many studies have shown that the adsorption of PFSAs (sulfonate group) on typical surfaces such as ACs or AERs is more favorable than that of PFCAs (carboxylate group), even though some PFCAs were more hydrophobic [53, 161]. More specifically, a study of multi‐PFAS adsorption on AER revealed the following trend: PFOS > PFHxS > PFOA > PFBS > PFHxA > PFBA. A similar trend was observed in adsorption on carbon nanotubes [53]. If hydrophobicity was the main driving factor, the removal capacity should follow the trend of $\log K_{\mathrm{ow}}$ (i.e. PFOS > PFOA > PFHxS > PFHxA > PFBS > PFBA). Instead, a trade‐off appears between hydrophobicity and headgroup nature.

Additionally, in realistic environments, PFAS coexist with other contaminants such as heavy metals, surfactants (e.g. cetyltrimethylammonium bromide and sodium dodecyl benzene sulfonate), and organic compounds (e.g. ethanol and hydrocarbons). These compounds participate in collective adsorption [126, 139, 157, 158, 159], which can enhance or reduce PFAS adsorption. In addition, both the adsorbent type and the physical interactions underlying adsorption should be identified to assess the impact of co‐pollutants on PFAS adsorption. Similarly, the effect of surfactants on PFAS adsorption depends on their concentration: both anionic and cationic surfactants appear to enhance adsorption but the effect is reversed once their concentration exceeds a certain threshold [158, 162]. Another study has examined the effect of organic compounds such as ethanol and 1,4 dioxane but the conclusions about their potential effect remain uncertain [157]. Nevertheless, this study has demonstrated strong competition between PFAS and Diethylene Glycol Butyl Ether (DGBE) – even at low copollutant concentrations (Figure 10b).

## 4 Fundamental and Practical Perspectives

### 4.1 Nomenclature and Assessment

#### 4.1.1 PFAS Definition

While PFAS are receiving increasing attention, establishing a reliable definition for these compounds remains a challenge that constrains the development of effective remediation strategies. In this review, the commonly accepted definition was used: a compound containing at least one – ${\mathrm{CF}}_{2}$ – or – ${\mathrm{CF}}_{3}$ – moiety [163]. Despite the 2021 report of the OECD on PFAS terminology [164], some ambiguities remain when assessing existing compounds – for example, the OECD provides an estimated number of 4730 PFAS [165], which contrasts with the 15 000 PFAS listed by the US EPA [166]. These discrepancies indicate the lack of consensus on a clear classification and census on PFAS definition and the need to standardize the terminologies for PFAS [167]. Several areas have been identified to achieve a coherent nomenclature: (1) development of a centralized database, (2) progress in *cheminformatic* tools for automated and structure‐based PFAS characterization/categorization, (3) identification and characterization of PFAS‐containing polymers, and (4) inclusion of organofluorine compounds not currently defined as PFAS [168].

#### 4.1.2 Concentration Assessment and Monitoring

While analytical tools exist for detecting and quantifying PFAS in natural environments, they can usually be classified as (1) targeted and (2) non‐targeted detection techniques. The methods belonging to the first class (e.g. Liquid Chromatography–Mass Spectrometry) are specific as they focus on the identification and quantification of a specific list of PFAS compounds. The second class encompasses methods that are less specific as they aim to detect a broader PFAS range, only providing an overall PFAS concentration (including compounds that were not in the list or unknown ones). While common analysis methods for targeted PFAS only allow the quantification of up to 200 compounds – a number that largely underestimates existing compounds – non‐targeted methods have allowed the detection of many more PFAS, including numerous unidentified compounds [169, 170, 171, 172]. Using Total Oxidizable Precursors and clustering analysis (non‐targeted detection methods), it was shown that emerging PFAS such as 6:2 FTAB, 8:2 FTAB or transformation intermediates (e.g. 5:3 FTCA) contribute to the compounds detected in sediments. Moreover, substantial contributions were found to arise from unidentified pre‐PFAAs (i.e., precursors of PFAAs) stemming from urban or industrial activities [173]. These results highlight the need to use complementary methods for analyzing environmental samples and to identify unattributed precursors (using suspect screening or non‐targeted screening). Because targeted strategies do not quantify all existing compounds, some authors have developed alternative approaches such as adsorbable organic fluorine (AOF), extractable organic fluorine (EOF), and total fluorine (TF) methods to quantify the “total amount of PFAS” [174]. Even if AOF and EOF successfully detect PFAS in complex media, they do not provide an exhaustive compound list. In contrast, the TF method provides better detection capacity even if it does not allow distinguishing organic and inorganic fluorine compounds [175]. The need for fast and cost‐effective detection in complex environments has driven recent advances in sensing technologies [176]. In light of the strong influence of PFAS properties on their adsorption, the development of efficient adsorption‐based remediation strategies is limited by the lack of rapid and accurate PFAS detection and identification tools, highlighting the need for substantial advancements in this field.

### 4.2 New Technologies and Alternatives

#### 4.2.1 Emerging vs. Legacy PFAS

Over the past two decades, most studies have focused on anionic PFAS, particularly perfluoroalkyl acids (PFAAs), due to their widespread occurrence in drinking water and natural environments. However, recent studies indicate that environmental samples contain a broader diversity of PFAS in terms of charge and functional groups than expected [177]. In particular, an increasing amount of cationic and zwitterionic PFAS have been identified in recent analyzes [178, 179]. For instance, many new highly fluorinated compounds identified in the composition of aqueous film‐forming foams (AFFF) raise concerns for remediation of contaminated sites. Similarly, analyzes of industrial wastewater treatment plants (WWTPs) have revealed the predominance of 6:2 FTAB, a zwitterionic PFAS whose adsorption behavior remains poorly characterized [180, 181]. While most adsorption studies mentioned in this review focus on anionic PFAS, several mechanisms remain relevant for other classes. In particular, the competition between electrostatic and hydrophobic interactions still governs the adsorption of cationic PFAS. Similarly, the competition between PFAS compounds and organic matter for adsorption on solid surfaces still holds for all PFAS classes. However, environmental factors such as pH or co‐existing ionic species may affect the adsorption of cationic and zwitterionic PFAS in different ways. In particular, despite their overall neutral charge – due to the presence of both positive and negative charges – zwitterionic PFAS can undergo both hydrophobic and electrostatic interactions. The contribution of collective adsorption mechanisms through the formation of surface aggregates might also play an important role for these compounds, but such mechanisms remain poorly explored in the current literature. Systematic studies involving various surface charges, PFAS functional groups and hydrophobicity are critically needed to identify the dominant adsorption mechanisms and assess the performance of conventional adsorbents for the broad class of PFAS compounds [177].

Another major challenge is the limited understanding of adsorption of short (with 4 to 6 –

$${\mathrm{CF}}_{2}$$

– moieties) and ultra‐short chain (with less than 4 – ${\mathrm{CF}}_{2}$ – moieties) PFAS. While short and ultra‐short chain alternatives are increasingly used due to regulatory constraints on long chain legacy PFAS [182, 183], most of the literature only concerns legacy PFAS. A recent assessment reported that only 50% of the existing literature concerns short chain PFAS, while only 33% characterizes ultra‐short chain ones – most of these studies focus on a single compound (PFBA) and do not provide estimates of the adsorption capacity [184]. Furthermore, short‐chain PFAS generally exhibit higher solubility and weaker hydrophobic interactions, leading to lower adsorption affinity and stronger competition with co‐existing species. As a rapid shift in regulations is expected in the next years, the development of novel technologies targeting these emerging PFAS is urgent.

#### 4.2.2 Conventional vs. Newly Developed Adsorbents

Owing to their low production cost and broad availability , conventional adsorbents such as activated carbons (AC) or ion exchange resins (IER) have been widely studied and commercialized for PFAS removal [185]. However, their performance remains limited by poor selectivity and reduced affinity toward emerging PFAS [186]. In this context, emerging adsorbents have been developed to overcome these limitations. Most of these materials can be classified into carbon‐based, mineral‐based, and polymer‐based adsorbents [186].

Carbon‐based materials, including graphene, graphene oxide, and carbon nanotubes combine high surface area, good chemical stability, and relatively low production costs [187]. However, their low selectivity toward PFAS compounds, particularly short‐chain species, limits their performance under realistic conditions due to strong competition with natural organic matter (NOM) [188]. Mineral‐based adsorbents offer the advantage of natural abundance and low cost. However, their negative surface charge under environmental conditions limits the adsorption of anionic PFAS. To overcome this challenge, surface‐functionalization is commonly employed. For instance, amine‐functionalized silica exhibits a positive surface charge that enhances electrostatic interactions with anionic PFAS. Clay‐based materials can also be modified with cationic polymers or nutrients (for detoxification of drinking water and food) [189]. Selectivity toward PFAS over other substances can also be enhanced with the use of fluorinated functional groups. Metal–organic–frameworks (MOFs) have also received significant attention in the past years. These nanoporous crystalline materials, made of metal nodes and organic linkers, exhibit a highly tunable nanoporous structure, large specific surface area, pore size and volume [190]. As a result, they offer high adsorption capacities (up to several hundreds of mg/g for PFAAs) and rapid kinetics (reaching equilibrium uptake in less than 1 min [191]). In addition, specific mechanisms such as acid‐base interactions between a PFAS and the framework can further enhance adsorption. Recent studies also report promising regeneration performance for this material, with adsorption capacities remaining close to 100 % over multiple adsorption/desorption cycles [76]. Similarly, covalent‐organic‐frameworks (COFs) are crystalline, porous polymers that offer tunable structure and surface chemistry [138]. Their adaptable design allows optimizing the spatial arrangement and ratio of hydrophobic and electrostatic interactions. However, their practical implementation is often limited by their powdered form, which complicates separation from treated water. The development of magnetic COFs has shown promising results to address this issue [192]. Polymer‐based adsorbents constitute another promising class of materials due to their tunable selectivity. In particular, the incorporation of specific functional groups or cross‐linkers enables targeted interactions with various PFAS types. For instance, negatively charged cross‐linkers have proven efficient in removing zwitterionic PFAS, while positively charged ones allowed capturing both short and long chain anionic compounds [193].

The performance of the emerging materials above often relies on surface functionalization. Amine groups enhance the adsorption of short and ultra‐short chain anionic compounds (which are typically poorly retained by conventional adsorbents), while fluorinated groups improve selectivity toward PFAS over competing pollutants and organic matter [194]. Despite these advances, several challenges remain for large‐scale applications. The long‐term stability of chemically modified adsorbents (MOFs and COFs in particular) and the impact of pH and temperature variations is not yet fully understood [186]. Additionally, many synthesis and functionalization methods rely on highly technical procedures and potentially hazardous chemicals, increasing both economic and environmental costs. For instance, amine‐containing materials raise sanitary concerns due to the potential release of nitrosamine precursors in drinking water [195]. Before practical implementation of these novel technologies, further investigation of regeneration strategies is also required. For instance, the high thermal stability of carbon‐based materials enables thermal regeneration techniques, which have proven to be highly effective, with a reported PFAS release and degradation of 99% in some cases [196]. However, such processes require high energy consumption and very high temperature (> 700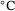 [197]), which limits their applicability to other classes of adsorbents. As mentioned previously for MOFs, chemical regeneration methods represent another promising approach for PFAS desorption, with desorption efficiency reaching 90% over several cycles [198]. Nevertheless, the solvents currently used in these processes raise environmental concerns, highlighting the need for more sustainable and cost‐effective solvents.

Finally, while surface chemistry plays an essential role in improving the affinity and the selectivity of solid surfaces toward PFAS, other aspects could be considered to design efficient adsorption‐based remediation techniques. For instance, performing several adsorption steps with adsorbents exhibiting different pore sizes could improve the removal of both short and long‐chain PFAS, while avoiding pore blockage. Indeed, as discussed in Section 3, large pore sizes are generally more favorable for the adsorption of long‐chain PFAS due to their lower solubility and higher affinity toward hydrophobic surfaces. Instead, only short‐chain compounds can access small pores through diffusion within nanoporous materials. Furthermore, designing efficient removal strategies in dilute regimes requires a precise knowledge of the mechanisms driving the adsorption process. In particular, the amphiphilic nature of PFAS compounds plays an important role as their adsorption may be driven by cooperative mechanisms (through the formation of surface aggregates) instead of single‐body interactions with the surface [93]. Thus, even when surface affinity is limited, cooperative effects may yield high removal efficiency in dense regimes. However, at very low concentration, cooperative adsorption mechanisms are strongly reduced, which limits the removal efficiency of the solid surface. This aspect is particularly relevant for PFAS remediation as even the small PFAS concentrations found in natural waters and drinking water sources represent an environmental and sanitary risk [199, 200]. The design of adsorption‐based remediation strategies therefore requires a precise knowledge of the driving adsorption mechanism to avoid overestimating the adsorbed amount in environmentally relevant concentration regimes.

### 4.3 Adsorption Experiments and Metrological Aspects

#### 4.3.1 Physical and Chemical Characterization

Developing analytical methods to determine the physical and chemical properties of PFAS compounds is essential for understanding and predicting their behavior in natural environments. Such characterization is needed to derive physicochemical models for multi‐solute systems and/or for adsorption in complex media subjected to pH, temperature, and concentration variations. However, many challenges remain in this field. As highlighted by several authors [63, 64], previous experimental assessments of PFAS properties did not provide reproducible results. For instance, large discrepancies have been reported for the critical micelle concentration (CMC) of PFOA, with values ranging from 45.54 to 15696 mg/L depending on the experimental conditions [14, 201, 202, 203, 204]. Beyond the strong variability for the few compounds whose CMC has been measured, the solubility and aggregative behavior of many PFAS compounds have not yet been characterized, despite their central role in evaluating PFAS adsorption. Similarly, PFAS hydrophobicity (e.g. as assessed via the octanol‐water partition coefficient) is essential to quantify the role of hydrophobic interactions in adsorption, but it has not been measured yet for most PFAS compounds. Furthermore, considering the essential role of the dissociation state of PFAAs in determining the nature of adsorption forces, the acidity constant $K_{a}$ must be accurately evaluated. However, it has not been well established for most acidic PFAS [205]. In this context, considering the wide diversity of PFAS, Murillo et al. have successfully applied predictive methods to estimate PFAS acid dissociation constant [116]. Such methods pave the way for fast and systematic PFAS characterization. The strong variability observed in PFAS properties can be attributed to differences in the experimental conditions under which the measurements were performed. While it illustrates the sensitivity of PFAS to environmental conditions, it also highlights the need for a precise characterization of the conditions under which adsorption occurs in order to accurately identify the underlying adsorption mechanisms. Finally, to improve our global understanding of PFAS adsorption under realistic conditions, it is essential to account for the temporal evolution of environmental factors such as fluctuating temperature and pH. Temporal variations significantly affect adsorption kinetics and can modify the final equilibrium state. In particular, slight changes in pH modify both surface charge and the dissociation state of PFAAs, while temperature variations influence adsorption by modifying the balance between energetic and entropic contributions.

#### 4.3.2 In‐Lab versus Practical Conditions

Fundamental research on PFAS adsorption only considers ideal – under lab – conditions which are difficult to extrapolate to realistic conditions. For example, although the estimated PFAS concentration in natural environments ranges from a few ng/L to a few dozens of μg/L (with a maximum regulatory level of a few ng/L) [206, 207], most instruments have a sensitivity threshold going down to a few μg/L [117]. Therefore, it is difficult to anticipate how adsorption tests and remediation techniques transfer to practical situations. Developing PFAS filters working for concentrations going from 3 to 6 orders of magnitude larger than in real media remains questionable. In addition, studies including the complex composition of water matrices are needed to establish proofs of concept [208]. Such studies must compare adsorption experiments in simple water matrices and in realistic environments, as it is necessary to verify whether or not PFAS adsorption in simple matrices follows classical models. Any deviations with respect to these models would allow identifying additional phenomena relevant to more complex media.

#### 4.3.3 Adsorption Equilibrium and Finite Size/Scaling Issues

**FIGURE 11:**
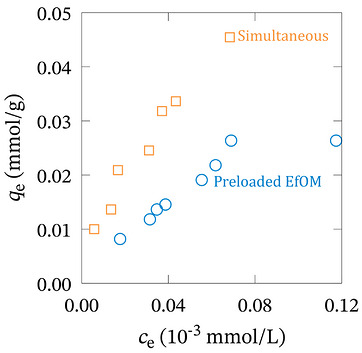
Adsorption isotherm of PFOA on PAC for two experiments [154]. The orange squares correspond to the amount of PFOA that was adsorbed upon its introduction simultaneously with EfOM in the solution. The blue circles correspond to the adsorbed amount of PFOA when introduced after letting EfOM adsorb and equilibrate.

PFAS adsorption is a very slow process that often takes hours to reach equilibrium. In this context, assessing adsorption thermodynamics requires conducting experiments over long periods to avoid inconsistencies. As an illustration, adsorption isotherm measurements conducted over 7 days have shown lack of coherence due to out‐of‐equilibrium conditions (Figure 11) [154]. The effect of EfOM was assessed during two different experiments: (1) simultaneous introduction of PFOA and EfOM in a solution before measuring the adsorbed amount on PAC after 7 days, and (2) introduction of PFOA in a solution after allowing EfOM to preload the surface for 14 days before evaluating the adsorbed amount. Even if the adsorption kinetics are expected to vary between those two experiments, the equilibrium adsorption isotherms should be equivalent. This double experiment was performed to illustrate a common issue with adsorption experiments on complex compounds in liquid solutions; either the measuring method does not allow for accurate adsorption isotherm assessment or the time is insufficient to reach equilibrium. The current methods for interpreting kinetic experiments also raise some concerns. Since conventional models assume that bulk concentration c remains constant, they fail to describe PFAS adsorption kinetics. Instead, depletion effects (i.e. variation of c upon adsorption) should be included – especially for small initial c.

#### 4.3.4 Adsorption Under Transport

Contamination and depollution are complex processes occurring under dynamic/kinetic conditions. Therefore, studying PFAS adsorption under flow is essential to evaluate their behavior in realistic conditions. However, it is often difficult to dissociate adsorption kinetics and transport during experiments [209]. In this context, numerical tools such as lattice Boltzmann methods are promising in characterizing these processes before experimentally studying their coupled dynamics [210].

### 4.4 Unconventional Thermodynamics and Kinetics

#### 4.4.1 Complex Adsorption Modeling

To date, over 100 adsorption models have been published to describe surface adsorption [211]. This plethora involves one, two, or more parameters – depending on the complexity of the model. While only few models possess physical foundations, many researchers have employed semi‐empirical models that successfully fit experimental curves, but do not provide any insights on the physical system and the adsorption mechanisms. For instance, the Freundlich model does not converge to Henry's law at low c so that it cannot serve as a reliable tool to interpret the underlying adsorption processes. Furthermore, these models often rely on conflicting hypotheses but are simultaneously used for comparison. It is for instance the case of many studies using both the Langmuir model which assumes a homogeneous surface and the Freundlich model that assumes a heterogeneous surface. More generally, the key hypothesis of a model should be carefully evaluated to ensure consistency with the physical system that is studied, before using it for fitting purposes. Additionally, in the light of the key parameters identified in this review (i.e. chain length, functional group, surface chemistry and topology, pH, co‐existing ions and organic matter), the choice of an appropriate adsorption model strongly depends on the adsorbent and adsorbate properties as well as on the solution chemistry. In particular, for short and ultra‐short chain PFAS that are relatively soluble, simple adsorption models neglecting lateral interactions may provide a sufficient description of adsorption thermodynamics. In contrast, for long‐chain PFAS undergoing significant hydrophobic interactions, models accounting for lateral interactions become necessary as adsorption may be driven by cooperative effects rather than by strong affinities toward the adsorbent only [93]. Furthermore, in complex water matrices containing co‐existing species (e.g. ions, co‐pollutants, or organic matter), multi‐component models should be systematically considered. More generally, as emphasized throughout this review, PFAS adsorption is governed by a combination of solubility, surface chemistry, and solution chemistry. In most existing models, these contributions are implicitly captured through the enthalpy of adsorption appearing in the adsorption constant ($k_{H}$ for Henry model and $k_{L}$ for Langmuir model). Developing a unified thermodynamic framework therefore requires making these contributions explicit and linking them to measurable physicochemical parameters. From a statistical physics perspective, a possible way for constructing such a framework would be to determine a functional form of the Hamiltonian associated with both the adsorbing surface and the adsorbed molecules that explicitly includes measurable parameters such as pH level, solubility, and ionic strength. For instance, in lattice gas models describing electrostatically driven adsorption mechanisms [92, 93], both the number of adsorbing sites and the single‐body interaction energy between the surface and the adsorbate could be expressed as functions of pH level and solubility. As a first step, systematic measurements of both the enthalpy of adsorption (i.e. the energy associated with the adsorption process) and the adsorption capacity (i.e. the number of available sites) could be performed over a range of pH values and/or solubility levels. These measurements could then be used to fit adsorption isotherms derived from the Hamiltonian of the system. Finally, factors such as pore/surface morphological and topological disorders could be included to better describe adsorption mechanisms on realistic surfaces [212, 213].

#### 4.4.2 Consistent Kinetic Modeling

While adsorption kinetics is essential, it is also important to understand the impact of other dynamical phenomena (e.g. diffusion, advection). Evaluating the characteristic time associated with each of these dynamics is crucial to determine the rate‐limiting process. In this context, any inconsistency between the thermodynamic model describing adsorption equilibrium and the kinetic model describing its underlying mechanisms provides valuable information. PFAS adsorption isotherms can often be fitted against a Langmuir model while the corresponding kinetic data obey a different underlying kinetic model [104]. When such departure is observed, other factors like reservoir depletion or adsorbent heterogeneities (which are neglected in the Langmuir model) are involved. For instance, static adsorption experiments can help determine whether saturation and/or depletion effects occur and, hence, guide the choice of appropriate kinetic modeling. In this context, while a plateau in the adsorption isotherm is often associated with surface saturation, comparison between the initial and final bulk concentrations allow probing depletion effects. While fitting of the kinetics can often be achieved with a first‐order (n=1) or second‐order (n=2) model, it is not always sufficient to conclude on the nature of the adsorption process. In fact, the validity of the second‐order model has only been established for early adsorption times and not for the entire adsorption duration [102].

The statistical treatment of experimental data is another major aspect to be considered when studying adsorption kinetics [214]. A study of the mathematical expressions used for statistical assessment of the best‐fitting mode showed that the classic linearization of the kinetic equations leads to comparing variables that are not equivalent [56, 215]. Specifically, the linearized first‐order kinetics are expressed in terms of $y_{1}\left(t\right)=log\left[q_{e}-q\left(t\right)\right]$ while the linearized second‐order kinetics involve the variable $y_{2}\left(t\right)=t/q\left(t\right)$. This difference leads to indicators which are not directly comparable between the two models [214]. Additionally, kinetic data are often unbalanced as very few data points are usually gathered before equilibrium, while many data points are collected at the plateau when adsorption equilibrium is reached [216]. In this context, the paper by Simonin provides some insightful recommendations about adsorption kinetics modeling [214]. In particular, the author highlights the importance of collecting a sufficient number of data points during the transient regime to ensure statistically robust parameter estimation. Furthermore, selecting a proper linearized expression for kinetic modeling in combination with some physical indicators can help to determine the relevant adsorption kinetics at play. For instance, the author suggests that a dependence of the kinetics on the adsorbent particle size indicates diffusion‐limited adsorption processes, which should guide the choice of the corresponding physical model used to describe the adsorption kinetics.

## 5 Conclusion

In this state of the art, we covered the fundamental principles of PFAS adsorption onto solid surfaces by reviewing the abundant literature on the topic. By considering both thermodynamic and kinetic aspects of these complex phenomena, we presented the ins and outs of PFAS adsorption onto natural and synthetic adsorbents – including active carbons, silicates/minerals, resins, etc. – while taking into account the role of parameters such as pH, salinity and the presence of other adsorbing species (ions, organic matter, etc.). First, we presented the main intermolecular interactions responsible for adsorption, and we detailed the resulting complex mechanisms – including specific PFAS effects that can be observed when considering the adsorption of these compounds. In this section, we also discussed the fundamentals of adsorption thermodynamics and kinetics onto solid surfaces. By adopting a fundamental physical chemistry standpoint, we focused on physical models which can be derived from sound theoretical considerations. Second, we introduced and reviewed the specificities of PFAS adsorption by considering the role of their rich physicochemical behavior. Specifically, despite many studies showing more favorable adsorption for PFSAs over PFCAs, the physical justification for such a trend remains to be understood and the role of the functional group size, polarizability and charge localization should be further investigated. We also addressed the impact of the adsorbent surface properties and how the surrounding chemical and thermodynamic conditions – e.g. pH, salinity, ionic strength, temperature – as well as the coadsorption and competitive adsorption with other compounds modify the behavior of PFAS near solid surfaces. Finally, in the last part of this review, we provided perspectives by identifying major issues that remain to be overcome to model and decipher PFAS adsorption on solid surfaces and in porous materials. In the specific context of PFAS depollution, we believe that the present review provides a reliable picture of PFAS adsorption onto solid surfaces. Yet, starting from nanoporous adsorbents, significant research efforts are still needed to understand the parameters and descriptors that govern PFAS adsorption. In particular, major gaps remain in the identification, quantification, and physicochemical characterization of PFAS. Addressing these challenges requires the development of analytical techniques with sufficiently high resolution and reproducibility. Additionally, a full characterization of adsorbent performance both in lab and under practical conditions is required. The first aspect, which could be investigated by performing adsorption experiments under controlled experimental conditions, would allow identifying the adsorption mechanisms (adsorption driven by surface affinity/low solubility or by cooperative effects, limiting factors for the kinetics (reservoir depletion, surface saturation or mass transfer mechanisms), etc.). The second aspect is necessary for evaluating the performance of the adsorbent under realistic conditions, including the presence of co‐existing species, variable chemical conditions, and fluctuating temperature. Long‐term stability, regeneration, and performance under dynamic conditions should also be carefully investigated. Overall, the development of efficient PFAS remediation techniques will require the integration of experimental and theoretical approaches within a robust and consistent thermodynamic framework to predict the adsorption of PFAS compounds in complex environments.

## Conflicts of Interest

The authors declare no conflicts of interest.

## Data Availability

The data that support the findings of this study are available from the corresponding author upon reasonable request.
